# Toll-like Receptor Mediation in SARS-CoV-2: A Therapeutic Approach

**DOI:** 10.3390/ijms231810716

**Published:** 2022-09-14

**Authors:** Abdul Manan, Rameez Hassan Pirzada, Muhammad Haseeb, Sangdun Choi

**Affiliations:** 1 Department of Molecular Science and Technology, Ajou University, Suwon 16499, Korea; 2 S&K Therapeutics, Ajou University Campus Plaza 418, 199 Worldcup-ro, Yeongtong-gu, Suwon 16502, Korea

**Keywords:** TLR, immune system, inflammation, antiviral, SARS-CoV-2

## Abstract

The innate immune system facilitates defense mechanisms against pathogen invasion and cell damage. Toll-like receptors (TLRs) assist in the activation of the innate immune system by binding to pathogenic ligands. This leads to the generation of intracellular signaling cascades including the biosynthesis of molecular mediators. TLRs on cell membranes are adept at recognizing viral components. Viruses can modulate the innate immune response with the help of proteins and RNAs that downregulate or upregulate the expression of various TLRs. In the case of COVID-19, molecular modulators such as type 1 interferons interfere with signaling pathways in the host cells, leading to an inflammatory response. Coronaviruses are responsible for an enhanced immune signature of inflammatory chemokines and cytokines. TLRs have been employed as therapeutic agents in viral infections as numerous antiviral Food and Drug Administration-approved drugs are TLR agonists. This review highlights the therapeutic approaches associated with SARS-CoV-2 and the TLRs involved in COVID-19 infection.

## 1. Introduction

Toll-like receptors (TLRs) are central mediators of the innate and adaptive immune responses. The immune system exhibits a defense mechanism for the host against pathogenic materials (exogenous and/or endogenous) at the cellular level [1]. Pattern recognition receptors (PRRs) including DNA sensors, RIG-1-like receptors, and TLRs are part of the innate immune system that protects against microbial infection. PRRs recognize conserved pathogen-associated molecular patterns (PAMPs) from microbes and endogenous danger-associated molecular patterns (DAMPs) produced by necrotic cells [2]. PAMPs are derived from viral, bacterial, parasitic, and fungal pathogens. The chemical nature of PAMPs recognized by TLRs varies greatly among organisms. In phylogenetics, TLRs are considered the most ancient class of PRRs. A large number of TLRs have been reported across a wide range of vertebrate and invertebrate species. The signaling pathways and adaptor proteins related to TLRs are evolutionary conserved, from Porifera to mammals. Moreover, similar domain patterns can be observed in most TLR homologs [3,4].

Viruses are responsible for initiating innate immunity through TLRs. Viruses, via a combination of small and unique proteins, not only escape the innate immune system but also destabilize the paybacks of the virus [5]. Similar to other pathogens, viruses are sensed by TLRs. Some viruses encode unique proteins that target TLR signaling. The hepatitis C virus encodes proteins that inhibit TLR-mediated signaling such as NS5A and protease NS3/4A [6,7], which inhibits MyD88 and cleaves TIR-domain-containing adapter-inducing interferon-β (TRIF), respectively. Moreover, the two vaccinia virus proteins have been reported as inhibitors of the TLR system; for example, A52R was observed to inhibit TLR-mediated NF-κB activation by targeting IRAK2 [8], whereas A46R exhibited a connection with TLR signaling downregulation by employing Toll-interleukin-1 receptor (TIR) domain-containing adaptors [9]. Intracellular TLRs not only sense viral and bacterial nucleic acids, but also identify self-nucleic acids in cellular abnormalities such as autoimmunity [10].

**Figure 1:**
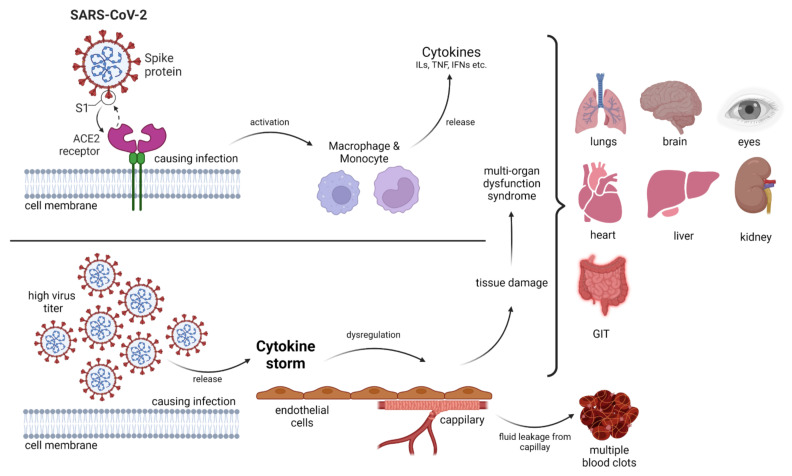
An overview of the SARS-CoV-2 infection pathway. During viral infection, immune cells are activated and release several cytokines as required for the biological system. A high virus titer is associated with a cytokine storm, and such dysregulation in the body of the patient may lead to multi-organ dysfunction syndrome. GIT—gastrointestinal tract.

A novel single-stranded RNA (ssRNA)-containing virus causes coronavirus disease (COVID-19), also referred to as severe acute respiratory syndrome coronavirus 2 (SARS-CoV-2), which became a pandemic after the first case was identified in Wuhan, China in December 2019. With the spread of COVID-19, the pandemic poses a global challenge [11,12]. From a clinical point of view, the virus has various manifestations ranging from patients becoming critically ill with acute respiratory distress syndrome to asymptomatic infection. In the intensive care unit, multiorgan support therapy has been essential in almost every case of COVID-19 (Figure 1). The critical disease stage is typically observed at 7–10 days of clinical infection [11,13]. Hyperinflammatory outcomes (cytokine storm) are mainly associated with clinical impediments and mortality [14]. A possible treatment methodology in the form of vaccines is being employed for the prevention of SARS-CoV-2 infection, but there is no operative therapeutic treatment option available. Consequently, exploring new drug targets is necessary. One of the most important molecular targets is TLRs. The interaction of the SARS-CoV-2 spike glycoprotein with TLR and the enhanced expression of genes associated with TLR signaling could indicate the possible involvement of these tiny molecular machines and their inflammatory cascades [14].

Structurally, TLRs are type I transmembrane (TM) proteins with three distinct domains including an extracellular domain (ectodomain) that contains tandem copies of leucine-rich repeats, a single-pass TM as well as a cytoplasmic TIR downstream-signaling domain. TLRs experience either homodimerization or heterodimerization when encountering PAMPs and/or DAMPs, and adaptor proteins are employed; subsequently, a complex cellular event of downstream signal transduction is initiated, leading to the expression of inflammatory cytokines and interferons (IFN) that is observable at the molecular level [2]. The underlying TLR signaling cascades have been elucidated using structural, genetic, biochemical, and in silico methodologies [15].

Downstream signaling is made possible by the presence of cytosolic TIR domain-adaptor proteins such as TRIF (also known as TICAM1), TRAM (TICAM2), MyD88, and MAL [5,16]. The involvement of TLRs with TIR adaptors leads to the activation of cytosolic signaling complexes including IRAK and TRAF proteins. These entities are responsible for the activation of transcription factors such as IRF and NF-κB. This executes the synthesis of type I IFNs and proinflammatory cytokines [16]. IRF7 is essential for IFN-α synthesis, NF-κB is necessary for TNF and IL-6 induction, and IRF3 and NF-κB are required for IFN-β production [17].

By neutralizing internal and/or external threats administered by TLRs, the innate immune system makes defensive contributions to the survival of the host biological system. However, dysregulation and/or overactivation of this system leads to various disorders such as inflammation, cancer, and autoimmunity [18,19,20].

## 2. Structure of Coronavirus

SARS-CoV-2, a member of the β-coronavirus genus in the family Coronaviridae, has an envelope and positive-sense ssRNA genome of 29,891 nucleotides, encoding circular nucleocapsid proteins with 9860 amino acid residues [21]. The viral particle size ranges from 80 to 220 nm. Overall, 10 open reading frames (ORFs) have been identified in its genome to date (approximately 26–32 kb). The first ORF (almost 2/3 of the viral RNA) encodes polyproteins 1a (ORF1a) and 1b (ORF1b) [22]. Furthermore, these ORFs are cleaved by proteases into 16 nonstructural proteins (NSPs) that are responsible for genome replication and transcription [23]. Structural proteins (SPs) are encoded by the remaining ORFs [24,25]. The main SPs and NSPs of SARS-CoV-2 are summarized in Table 1 and Table 2, respectively. The name coronavirus is derived from the appearance under the electron microscope, in which the presence of crown-like spikes on the envelope resembles the corona of the sun [26]. SPs form the viral envelope that holds the RNA genome, while NSPs are expressed in host-infected cells but are not incorporated into virion infectious particles. These NSPs include various transcription factors and enzymes such as RNA-dependent RNA polymerase (RdRp) and hemagglutinin esterase (HE). Moreover, the virion employs enzymes such as RNA replicases and viral proteases to replicate itself [22,27,28,29].

Various SPs have been identified including the glycoprotein membrane (M), spike (S), small envelope (E), and nucleoprotein (N), and other accessory proteins. M-glycoprotein is the most abundant, spanning the membrane bilayer thrice [30]. S-glycoprotein (150 kDa) is a type-I TM protein on the outer surface of the virus and is responsible for the binding of the virus to host cell receptors (ACE2). The S protein amino acid sequence of SARS-CoV-2 exhibits 86% similarity to that of SARS-CoV [31]. The S protein consists of oligosaccharides bound to serine amino acids through *o*-glycosides. The three major segments of S protein are the ectodomain, TM, and intracellular regions. The intracellular domain comprises the membrane fusion subunit S2 (trimeric stalk) as well as a short tail part known as the receptor-binding S1 domain (RBD; three S1 heads) [32,33]. Protein–protein interaction (PPI) between the human ACE2 and SARS-CoV-2 S protein facilitates viral attachment as well as the cellular entry of coronaviruses; thus, small-molecule blockage of these PPIs is a more inspiring therapeutic approach than inhibition via antibodies [34]. The S1 subunit of the S protein enables ACE2-mediated virus attachment, whereas the S2 subunit facilitates membrane fusion. Specifically, asparagine, glutamine, serine, phenylalanine, and leucine residues present in the S protein boost ACE2 binding [35].

Moreover, N protein bound to nucleic acids is an important structural component of the virus, which is responsible for viral replication and cellular response to infection in the host cellular machinery [31] (Table 1). The N protein comprises a serine-rich linker region sandwiched between the N-terminal domain (NTD) and the C-terminal domain (CTD). These termini are crucial for viral entry and processing in host cells. The CTD regulates nucleocapsid formation and the NTD adheres to the viral genome in the form of orthorhombic crystals. Phosphorylation sites are also present in the linker region, which control its function [35]. In the case of SARS-CoV, the N protein enhances the activation of cyclooxygenase-2 (COX-2), resulting in the inflammation of pulmonary cells [36]. Moreover, the N protein interacts with the p42 proteasome subunit, which degrades the virion [37]. This also disables type-I IFN, which is responsible for suppressing the host immune responses produced by biological systems against viral infections [38]. The interaction of the N protein with heterogeneous nuclear ribonucleoproteins leads to increased viral RNA synthesis [39]. The N protein sequence of SARS-CoV-2 shows a 94.3% similarity to that of the SARS-CoV [31].

The smallest TM structural protein in coronaviruses is the E protein (Table 1), which comprises two different domains: the NTD (1–9 residues) as well as a hydrophobic domain (10–37 residues), with a chain at the terminal (38–76 residues) [40,41,42]. The E protein plays a crucial biological role, not only in the structural integrity of the virus, but also in host virulence [43]. The E protein sequence of SARS-CoV-2 shows a 96.1% similarity to that of SARS-CoV [31].

The M protein plays a crucial role in maintaining the shape of the viral envelope (Table 1). This function can be achieved by interacting with other viral proteins that exhibit PPIs [44]. The M protein is also known as the central organization of coronavirus proteins. The binding of E to M produces the virus envelope, and this interaction is sufficient for the synthesis and release of viruses [45,46]. The binding of M with S is an important event for the retention of the S protein in the endoplasmic reticulum–Golgi complex as well as its integration into new viruses [46,47]. Moreover, the interaction of N with M stabilizes the nucleocapsid (RNA–N protein complex) and the internal core of viruses, resulting in the completion of viral assembly [47,48]. The M protein amino acid sequence of SARS-CoV-2 exhibits a 96.4% similarity with that of SARS-CoV [31].

**Table 1:** The structural proteins (SPs) of coronaviruses and their physiological significance.

Sr. No.	SPs	PDB ID	Residues	Physiological Significance	Reference
1	E	7K3G	76–109	Virus assembly, morphogenesis, viral–host interaction, membrane permeability	[49]
2	M	8CTK	220–260	Virus assembly, protein interactions (M–M, M–S, M–N)	[50]
3	N	6VY0, 6YUN	422	Abundant RNA-binding protein, virion genome packaging	[51]
4	S	6VYB	1273	Main antigen component, triggers the host immune response	[52]

**Table 2:** The non-structural proteins (NSPs) of coronaviruses and their physiological significance.

Sr. No.	NSPs	PDB ID	Residues	Physiological Significance	Reference
1	NSP1	7K3N	180	Protein synthesis, prevents antiviral activity of host cells, degrades host mRNA	[53,54,55]
2	NSP2	7MSW	638	Genome replication, disruption of intracellular host signaling	[56,57,58]
3	NSP3 (Papain-like protease, PL_pro_)	7KAG, 6WEY, 6WUU, 7LG0	1945	Integral to viral replication, post-translational processing of the two polyproteins, suppresses host protein synthesis	[22,58,59]
4	NSP4	3GZF	500	Protects new replicated virions, replication and assembly of viral structures in host cell	[60,61]
5	NSP5 (3C-like protease, 3CL_pro_)	6LU7	306	Protein cleavage capacity (conserved feature)	[62,63]
6	NSP6	-	290	Induction of autophagosomes, inhibition of viral components to reach host lysosomes	[64,65,66]
7	NSP7	7JLT	83	Primase complex (NSP7-NSP8), hetero-oligomeric complex (NSP7-NSP8-RdRp), viral replication	[67,68,69]
8	NSP8	7JLT	198	Primase complex (NSP7-NSP8), hetero-oligomeric complex (NSP7-NSP8-RdRp), viral replication	[67,68,69]
9	NSP9	6WXD	113	RNA synthesis, carries viral RNA to the host cell, responsible for proliferation	[70,71,72]
10	NSP10	6ZPE	139	Cofactor activation for replicative enzymes, complex NSP10-NSP14, viral RNA proofreading	[73,74,75]
11	NSP11	-	13	Cleavage product of PP1a by 3CL_pro_/M^Pro^	[21,76]
12	NSP12 (RNA polymerase, RdRp)	6YYT	932	RNA polymerase activity	[29,77,78,79,80]
13	NSP13	6JYT	601	Helicase activity	[29,81]
14	NSP14	7R2V	527	Viral RNA methylation, viral RNA proofreading, methyltransferase activity	[73,82,83,84]
15	NSP15	6WXC	346	Endoribonuclease activity	[81,85]
16	NSP16	6WVN	298	Viral replication, immune response evasion Viral RNA methylation, methyltransferase activity	[84,86,87]

## 3. Overview of TLR Signaling

**Figure 2:**
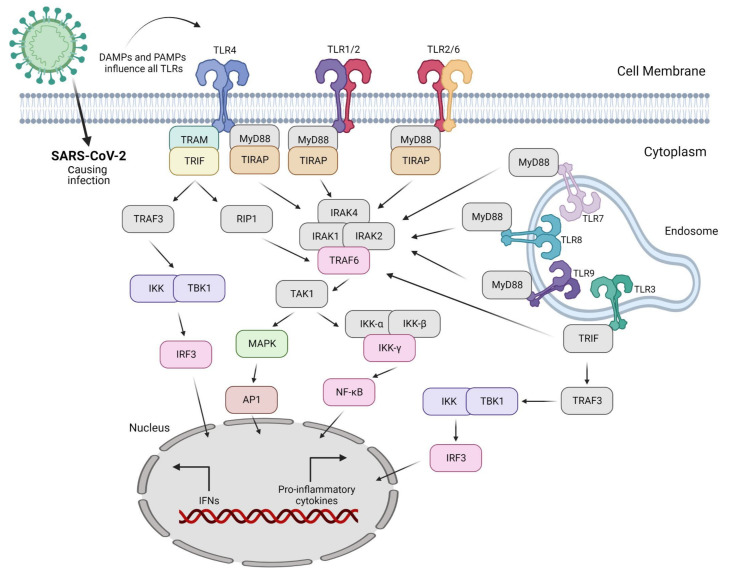
SARS-CoV-2 causes infection in the lungs mainly via DAMPs and PAMPs produced as a result of the action of nearly all Toll-like receptors (TLRs). Only TLRs involved in virus sensing and/or signaling are displayed here.

Invading pathogens stimulate the release of proinflammatory mediators in response to infection (Figure 1 and Figure 2). Signaling networks are necessary for the protection of the host against invading microorganisms. TLR signaling dysregulation plays a central role in the development and progression of infection. Inflammatory secretory molecules including chemokines, ILs, IFNs, and tumor necrosis factor-alpha (TNF-α) are part and parcel of TLR signaling, resulting in the modulation of cellular characteristics such as apoptosis, immune response, and proliferation [88,89,90]. Mitogen-activated protein kinases (MAPKs) and NF-κB are activated by TLRs. TLR3 and TLR4 are involved in the stimulation of IRF3. In contrast, IRF7 is triggered by TLR7–9 [91]. TLRs are stimulated by interactions with ligands to initiate an intracellular downstream signaling cascade, leading to activation of the host defense system [92].

The nature of the ligand and downstream adaptor molecules directs the TLR signaling cascade (Table 3). Two distinct pathways play critical roles in TLR signaling: MyD88-dependent and -independent pathways [93] (Figure 2). The former pathway employs all TLRs (except for TLR3), resulting in the biosynthesis of inflammatory cytokines [94]. In contrast, the latter pathway (also referred to as the TRIF-dependent pathway) involves TLR3 and TLR4, resulting in the expression of IFN-I [95]. In other words, the interaction of PAMP and PRR leads to the biosynthesis of proinflammatory cytokines as well as IFN-1, which is a cellular indication of the immune response [96]. Several negative regulators that enhance the activation of the innate immune response are involved in TLR-dependent signaling cascades. Hence, the overactivation of TLRs can lead to the interruption of immune cell homeostasis, resulting in the risk of inflammatory disorders [97]. Consequently, inhibitors (antagonists) targeting these receptors and/or cascades can serve as novel therapeutics to treat such disorders [98].

## 4. Role of Antiviral Drugs Employing TLRs

When a pathogen such as a virus invades, an antiviral immune response is evident in the host cells. Various conserved molecular patterns of PAMPs have been identified. As discussed above, TLRs are the key constituents of the innate immune system, and multiple TLRs (TLR1–4, TLR6–9) identify viral ligands [17,117,118,119]. With respect to their functional importance, TLRs might be potentially employed to treat not only inflammatory disorders but also viral diseases. This can be explained by a deep insight into the positive and negative mediators of TLRs [97,120]. TLR agonists lack accessory molecules but can mimic natural ligands; hence, they exhibit a low molecular weight and have potential for expanded pharmacokinetics and pharmacodynamics in comparison with the parent molecule. Moreover, TLR antagonists help to deal with autoimmune and inflammatory disorders by defeating unnecessary inflammation, resulting in an antibody- or cell-mediated response that suppresses disease progression [97,121,122].

Different approaches are employed by viruses in which they weaken their recognition by masking and/or increasing the dysregulation of mediators. Viruses disturb TLR signaling through their own mechanisms. Thus, TLRs are largely involved in the molecular interaction between viruses and host cells [5]. Various PRRs are engaged in the response to viral infection, which is also the case for TLRs. A thorough understanding of this interaction has facilitated the development of various strategies to limit viral infection including antiviral immunity as well as therapeutics [5]. Moreover, viral infection activates TLRs to increase cytokine levels, resulting in an antiviral innate immune response. The interaction between viruses and TLRs at every step of the signaling pathway plays an important role in developing effective antiviral therapies as well as in identifying novel molecular targets for the advancement in antiviral drugs [123]. The regulation of invasion, replication, and immune responses is a significant factor in viral pathogenesis [117]. Viral glycoproteins and NSPs released in the extracellular region are responsible for the stimulation of TLR2 and TLR4 due to their presence on the cellular surface [117,124,125]. In contrast, TLR3, TLR7/8, and TLR9, which are present in the endosomal compartment, contain viral double-stranded RNA (dsRNA) [126], ssRNA [114], and CpG DNA (unmethylated) [116], respectively.

TLR agonists have a positive effect on antiviral immunity and exhibit significant resistance against experimental infections [127,128,129]. The TLR–virus interaction involves a complex mechanism that is associated with the type of TLR as well as the type of virus. Moreover, multiple PRRs are required to initiate an immune response to various viral infections. Moreover, significant differences in TLR signaling have been reported between mice and humans. Therefore, therapeutic manipulation of TLRs requires an understanding of human cellular immunity [130]. Some examples are presented below.

TLR2 activation enhances the innate immune response to viral infections and can be used to treat viral respiratory diseases. Using the shock-and-kill strategy, immune cell recognition is enhanced and latently infected cells are eliminated [112,131]. TLRs can be used to reverse HIV-1 latency and trigger innate immune responses. In an evaluation of the effectiveness of SMU-Z1 (a novel TLR1/2 agonist), in addition to enhancing latent HIV-1 transcription (ex vivo), the NF-κB and MAPK pathways were also targeted in cells [131]. Latency-reversing agents have been employed for HIV reactivation, resulting in enhanced immune activation [112]. Dual TLR2/7 agonists were synthesized and characterized based on their latency-reversing ability, which were found to effectively reactivate the latency. TLR2 components reactivate HIV by NF-κB stimulation and the secretion of IL-22 (thereby enhancing the antiviral state and inhibiting HIV infection), whereas TLR7 components induce the secretion of TNF-α [112]. The activation of TLR2 in vivo has been assessed against rhinovirus infection [132]. Airway epithelial cells promote an extended immune response characterized by IFN-λ expression, NF-κB activation, and lymphocyte recruitment, resulting in a reduction in viral-induced inflammation and continued antiviral innate immunity [132].

TLR3 (the first identified antiviral TLR) in humans confers protective immunity against vaccinia virus (VACV) infection. In contrast, TLR3 is responsible for the detrimental effects of VACV infection in mice and TLR4 has the same effect in humans [133,134]. The recognition of dsRNA by TLR3 is further evidence of the role of TLRs in the antiviral response [119,126,135]. TLR3 signaling can be activated by a synthetic dsRNA agonist (a potent immune stimulant), resulting in protective immunity against multiple viruses including coronaviruses [136,137,138,139]. Viral-origin ssRNA sequences (rich in GU- and AU-) are detected by TLR7 and TLR8, which are functionally similar and only differ with respect to their expression patterns [113,130]. TLR7/8 expression is evident in dendritic cells, monocytes, and macrophages [140]. Additional examples are listed in Table 4.

## 5. Possible Molecular Interactions of SARS-CoV-2 with TLRs

SARS-CoV-2 is not only associated with viral illness but also with disorders of immunopathology. DAMPs and viral components act as TLR ligands for their overactivation. TLR4 (membrane-bound) and TLR3/7/8 (endosomal) play significant roles in the production of cytokine storms. The ssRNA of SARS-CoV-2 is recognized by TLR7/8, and after replication, the viral dsRNA is recognized by TLR3, which leads to TRIF-mediated inflammatory signaling [152]. The MyD88-dependent pathway (leading to overactivation of TLRs), related to the TRIF pathway, provides a possible link between SARS-CoV-2 and TLRs [153]. The production of type I (IFN-α and IFN-β) and type III [IFN-λ (1/2/3)] IFNs by TLRs is a significant antiviral feature that can be exploited for systematized viral control as well as clearance [117,119,154]. Type I and III IFNs perform the same function (despite their structural differences) in cellular signaling, although type III IFN receptors are primarily localized to the epithelial surface (airway epithelial cells) [155]. Cytokines (type III IFN) bind to their receptors, and the signal cascade is initiated by the JAK/STAT pathway, leading to the formation of IFN-stimulated genes [156]. Activation of the JAK/STAT pathway induced by TLRs may lead to macrophage activation syndrome [157]. Virally infected cells are killed by activated dendritic cells, natural killer cells, and macrophages stimulated by IFN [158]. SARS-CoV-2 infection results in higher levels of chemokines and proinflammatory cytokines in the blood [159,160]. These biological conditions lead to host cell death and organ injury [161]. Hence, the synthesis of DAMPs amplifies inflammation by TLR binding via the MyD88-dependent pathway. Elevated TLR stimulation, signaling cascades, and NF-κB may influence the severity of COVID-19 [153]. The nutritional profile has a basic influence on immunity. Compounds with immunomodulatory, anti-inflammatory as well as antiviral characteristics can be helpful against SARS-CoV-2 infection [162]. Various studies have suggested encouraging results in the case of nutraceuticals [163,164]. Compounds including astaxanthin, curcumin, glycyrrhizin, hesperidin, lactoferrin, luteolin, quercetin as well as resveratrol may inhibit and counteract the symptoms of COVID-19 [165,166,167,168,169,170,171,172].

Accordingly, IFN has been dynamically explored as a therapeutic target for COVID-19. This is because the release of type III IFN in the lungs could be responsible for the observed immunopathology of COVID-19 [173,174]. In contrast, type I IFN in combination with antiviral drugs has exhibited the opposite results including reduced systemic inflammation and viral clearance [173,174,175]. The synthesis of proinflammatory cytokines is associated with MyD88-dependent pathways, whereas the activation of type I and III IFNs is linked with the TRIF-dependent pathway [176,177] (Table 3). SARS-CoV-1 dsRNA and ssRNA are not detected by TLR3 and TLR7 and show some protective dodging mechanisms; hence, the same strategy could be used by SARS-CoV-2 [178,179]. The stimulation of TLRs by SARS-CoV-2 is responsible for activation of the inflammasome and the subsequent release of IL-1β and IL-6. Moreover, enhanced inflammasome activation is linked to non-promising consequences in patients with COVID-19 [180]. TLR2 signaling is activated by SARS-CoV-2 infection (Table 5). Thus, blocking of the signaling has been proposed as a potential target for the treatment of COVID-19 [181] because the strong effect of proinflammatory cytokines leads to disease severity through the activation of TLR2 [182]. In the context of infection with β-coronaviruses, MyD88, the TLR adaptor, has been reported to be a significant factor in the release of a large number of inflammatory cytokines [183]. SARS-CoV-2 interacts with various TLRs, directly or indirectly. Multiple interacting residues have been reported in the literature considering PPI and the design of agonists/antagonists. The interacting residues are based on experimental as well as computation studies. Only TLRs involved in virus sensing and/or signaling are displayed (Table 5).

Multiple TLR (2, 4, 7, 9)-deficient macrophages were infected with the mouse hepatitis virus. TLR2 deficiency resulted in the inhibition of TNF and IL6 expression as well as inflammatory cytokine genes. In contrast, other TLR deficiencies had negligible effects on these genes [182]. In the case of SARS-CoV-2, an inhibitor of TLR2 caused a noteworthy reduction in cytokine and chemokine release. This study demonstrated the role of TLR2 in sensing viral invasion upstream of MyD88 [182]. The TRIF pathway activated by TLR3 showed a protective response against Middle East respiratory syndrome (MERS)-CoV and SARS-CoV infections [184] (Table 3 and Table 5). Mice lacking TLR3, TLR4, and TRIF adaptor are exceedingly vulnerable to SARS-CoV and enhanced pulmonary infection, resulting in a risk of mortality [185]. Moreover, a role of TLR4 has been identified in the pathology of SARS-CoV-2, characterized by excessive inflammation in patients and activation of the inflammasome [186,187]. TLR4 inhibition in animal models has been shown to decrease lung injury by alleviating NF-κB pathway stimulation [188]. Viral infection and subsequent inflammation results in the production of DAMPs that act as ligands for TLR4. Heat shock proteins released from virus-infected cells act as TLR4 agonists [189]. TLR5 has been proposed as a target against SARS-CoV-2 in the development of drugs and vaccines [190].

During cytokine storms, elevated levels of IL-6 in the serum have been observed in patients with COVID-19 (Figure 1). TLR7 (activated by viral components) stimulates the MyD88-dependent pathway, resulting in the release of ILs and TNF-α, particularly IL-6 [191,192]. Structurally and phylogenetically similar receptors but different TLR7/8 agonists synthesize different cytokines [193]. ssRNA fragments in SARS-CoV-2 induced by TLR7/8 have been detected [194]. Whole-genome sequencing of SARS-CoV-2 in comparison with other coronaviruses (MERS-CoV and SARS-CoV) has revealed that TLR7 could be significantly involved in COVID-19 as the viral genome contains more ssRNA motifs that can bind to TLR7 [195]. Moreover, the TLR7 agonists imiquimod and imidazoquinolinone (with a role in TLR7activation) are under investigation as potential therapeutics against COVID-19. These drugs have been observed to decrease systemic inflammation and innate immune activation due to their antiviral effects [196,197]. RNA and DNA rich in unmethylated CpG islands can be recognized by TLR9. Both viral and mitochondrial DNA enriched in the same sequence are associated with inflammatory responses involving TLR9-mediated signaling. Moreover, the activation of p53 [198] and mammalian target of rapamycin (mTOR) is being considered as a therapeutic target against SARS-CoV-2. mTOR blockers are also associated with the MyD88 and TLR9 pathways [199].

**Table 5:** The interaction of Toll-like receptors (TLRs) with SARS-CoV-2 and other coronaviruses.

Coronaviruses	TLRs	Interacting Residues of TLRs	References
SARS-CoV-2	TLR2	Tyr323, Phe325, Val 348, Phe349	[182,200,201]
	TLR3	His39, His60, His108, Asn515, Asn517, His539, Asn541, Arg544, Ser571	[111,202,203]
	TLR4	Arg264, Glu266, Asp294, Tyr295, Tyr296, Thr319, Glu321, Lys341, Lys362, Gly363, Gly364, Arg382	[188,204,205]
	TLR7/8	Phe349, Tyr356, Gly379, Val381, Phe408, Asp555, Leu557, Gly584, Thr586	[114,206,207,208]
SARS-CoV	TLR3	His39, His60, His108, Asn515, Asn517, His539, Asn541, Arg544, Ser571	[136,185,203]
	TLR4	Arg264, Glu266, Asp294, Tyr295, Tyr296, Thr319, Glu321, Lys341, Lys362, Gly363, Gly364, Arg382	[185,205]
	TLR7/8	Phe349, Tyr356, Gly379, Val381, Phe408, Asp555, Leu557, Gly584, Thr586	[207,208,209]
MERS-CoV	TLR3	His39, His60, His108, Asn515, Asn517, His539, Asn541, Arg544, Ser571	[203,209]
	TLR4	Arg264, Glu266, Asp294, Tyr295, Tyr296, Thr319, Glu321, Lys341, Lys362, Gly363, Gly364, Arg382	[205,210,211]
	TLR7/8	Phe349, Tyr356, Gly379, Val381, Phe408, Asp555, Leu557, Gly584, Thr586	[207,208,209,212,213]

## 6. Promising Drug Targets in SARS-CoV-2

Possible and effective drug targets as well as therapeutic agents against SARS-CoV-2 have been suggested by various researchers worldwide [214]. For example, virulence factors, enzymes, host-specific receptors, and glycosylated-structural proteins have been identified in pathological conditions caused by the coronavirus [215]. Activators of transcription signaling pathways, proinflammatory cytokines, Janus kinase/signal transducers, and NSPs also play a crucial role in the pathology. Antiviral therapeutic strategies such as drug repurposing depend on chemical and molecular interactions between the host machinery and viral small molecules [215].

Low-molecular-weight molecules from plants (phytochemicals) have been tested for their antiviral activity. Compounds extracted from plants have been shown to exhibit antiviral activity against SARS-CoV in Vero cells. Lycorine was identified as the active ingredient of *Lindera aggregata*, and it has been suggested that the plant extract and lycorine can be a good option for the development of novel antiviral drugs [7]. In plants, secondary metabolites are produced by metabolic pathways, which are also referred to as phytochemicals [216]. These metabolites have been screened for their efficacy against microbes and viruses. Various phytochemicals have been shown to inhibit viral infection and replication [217]. Bioactive phytochemicals can improve and strengthen host immunity. For example, less vulnerability to infections and assistance in the stoppage of viral infections through host immune function have been observed with the treatment of vitamins A and C [218]. Various in vitro, in vivo, and in silico models using marine-derived natural compounds exhibiting promising anti-SARS-CoV-2 efficacy have been previously summarized [219].

Various proteins including ACE-2, RdRp, 3CLpro, PLpro, RBD, and cathepsin L could be operative therapeutic targets [67,198,220,221,222,223]. Although several molecules have been suggested as drug candidates, currently, there are no accessible operative anti-CoV mediators. Molecular interactions between ACE2 and SARS viruses are determinants of the initial infection. Hence, renin–angiotensin–aldosterone system (RAAS) inhibitors may modify ACE2 expression, resulting in reduced SARS-CoV-2 virulence. ACE2 (type I transmembrane-metallocarboxypeptidase enzyme) controls the effects of RAAS and is a key receptor for both SARS-CoV-1 and SARS-CoV-2, which facilitates entry into human lung cells through the S protein of the coronavirus [224,225,226,227,228]. Considering the complexity of the pathogenesis of SARS-CoV-2, clinically approved drugs that stimulate ACE2 may serve as operative anti-SARS-CoV-2 therapeutics [229]. ACE inhibitors (captopril) stimulate the ACE2/angiotensin (1–7)/receptor axis [230]. In animal models, treatment with angiotensin receptor blockers was shown to enhance ACE2 expression [231,232]. The ACE2–RBD complex is proteolytically regulated by type-2 transmembrane cellular serine protease (TMPRSS2), which leads to ACE2 cleavage and S protein activation [233]. The RBD (S protein) of SARS-CoV-2 contains more ACE2-interacting residues (Tyr473, Gln474, Cys488, Tyr489, Val524, and Cys525) than that of SARS-CoV, and is involved in loop formation. These mutations are evident in the sequence (RBD) of SARS-CoV-2 [234,235,236]. Moreover, two binding hotspot residues (Lys31 and Lys353) have been reported to be more sensitive to S protein binding. Lys31 and Lys353 formed salt-bridge(s) with Glu35 and Asp38, respectively, surrounded by a hydrophobic region [237]. Additionally, other studies support the development of promising ACE2 inhibitors for SARS-CoV-2 [220,238,239].

In the case of glycosylated S protein, membrane fusion inhibitors for the S2 subunit and antibodies (monoclonal) targeting the S1 subunit could be operative therapeutic mediators to treat coronavirus infection. Vaccine development has also been promoted against coronaviruses. Small-molecule inhibitors (SMIs) might be suitable for inhaled and/or oral administration, exhibit less mutation and strain sensitivity, are less immunogenic, and convenient. Novel drug-like SMIs (DRI-C23041 and DRI-C91005) have been identified. These SMIs inhibit the interaction between S protein and human ACE2 [34]. Moreover, griffithsin, a compound derived from red algae, adheres to the SARS-CoV-2 glycosylated S protein as well as to HIV [240]. Furin, a serine endoprotease, cleaves S1–S2 and may be suitable as an anti-SARS-CoV-2 agent [241]. Emodin, a *Rheum tangutica*-derived compound, not only inhibits the interaction between the ACE2 receptor and SARS-CoV-2 but also blocks SARS-CoV ORF3a [242,243]. Moreover, the host protease is employed by SARS-CoV-2 for the priming of the S protein. Camostat mesylate, an inhibitor of proteases, helps in the infection of lung cell lines [244]. Similar to the S protein, other structural proteins as well as NSPs have been highlighted as potential targets for the development of antiviral drugs. In the RBD, 14 different potent residues of the S protein have been identified that interact significantly with ACE2, resulting in the stability of the complex [245], while 15 significant residues in the RBD of the S protein have been reported in the case of the Omicron SARS-CoV-2 variant [246]. Both of these studies analyzed (in silico) natural compounds for their anti-SARS-CoV-2 bioactivity [245,246]. Additionally, terpenes (natural compounds) have been suggested as anti-SARS-CoV2 binding agents between the RBD and ACE2 receptor. Terpenes showed a strong affinity for RBD and inhibited its interaction with ACE2 [245].

Toremifene, a nonsteroidal selective estrogen receptor modulator, was found to block the viral replication of coronaviruses such as MERS-CoV and SARS-CoV [247] and the Ebola virus [248] by targeting viral membrane proteins. Hence, it has also become a potential candidate inhibitor of SARS-CoV-2 replication [248]. Moreover, a team of researchers proposed a region (residues) in the M protein for the development of novel drugs and/or peptides to block dimer formation [249]. The interaction of the M protein (heterodimer) with the S and E proteins (via PPIs) has been proposed by computational analysis, and key amino acids for the M–E complex (W55, F96, F103) and M–S complex (Y71, Y75) have been identified [250].

A high percentage of E protein is expressed inside the infected cells, which is responsible for viral assembly, maturation, budding, and proliferation [35,40,214]. The percentage similarity of the SARS-CoV-2 E protein sequence with that of other coronaviruses (96.1%) [31] demonstrates the potential for repurposing and/or development of pan-anti-corona drug candidates. Small molecules (phytochemicals) such as belachinal, vibsanol B, and macaflavanone E have been evaluated for the inhibition of E protein activity by in silico analyses [251].

The N protein exhibits essential activities such as proliferation of the virus as another important component, similar to other SPs. This provides a promising area for developing effective therapeutics to inhibit viral proliferation. For example, glycogen synthase kinase-3 (GSK-3), also known as serine/threonine protein kinase, is an important component of N protein phosphorylation. GSK-3 inhibitors inhibit N protein phosphorylation and result in damaged proliferation (infected lung epithelial cells) in SARS-CoV-2 in a cell type-dependent manner [252]. Candidate inhibitors of the N protein have been suggested by a screening method on a biochip platform using a quantum-dot (QD) RNA oligonucleotide. The novel anti-SARS potential of catechin gallate and gallocatechin has been identified. These two molecules (0.05 µg/mL) presented more than 40% inhibition activity on a QD-based RNA oligonucleotide system [253]. Computational analysis has suggested that zidovudine triphosphate is a potent inhibitor of the N protein of SARS-CoV-2 [254]. Based on an in silico approach, another repurposing study shed light on vanganciclovir, which is approved for treating patients with HIV and shows activity against N protein as well as the main protease [254].

SARS-CoV-2 depends on proteases of the Golgi apparatus to synthesize NSP1–16 in the host cell [27]. NSP3 [papain-like protease (PL_pro_)] is a multidomain protein and the largest protein in the coronavirus genome. Several regions of the NSP3 gene are involved in viral replication. NSP3 contains a SARS-unique domain that can attach to G-quadruplexes, which are guanine-rich non-canonical nucleic acid structures that are essential for SARS-CoV replication. SARS-CoV-2 NSP3 shows structural similarities [255]. By developing a protease assay and screening a custom compound library, two molecules (dihydrotanshinone I and Ro 08-2750) were identified to significantly inhibit PL_pro_ in protease. Additionally, the inhibition of viral replication was evaluated by an isopeptidase assay using cell culture [256]. Another protease, NSP5 (3CL_pro_), was identified as a primary target (similar to PL_pro_) for coronavirus drug discovery. Both of these targets are crucial and have conserved activity in the proteolytic processing of viral replicase polyproteins [257].

Coronaviruses encode two or three proteases that cleave replicase polyproteins. Many NSPs assemble into the replicase–transcriptase complex, which generates a reasonable environment for RNA synthesis and subsequent replication as well as the transcription of sub-genomic RNAs [258]. Replicase polyproteins 1a and 1ab are comprised of NSPs11 and 16, respectively [259]. M^Pro^ (the main protease), commonly known as NSP5, is employed for the cleavage of these polyproteins, exhibiting crucial events of viral assembly and maturation [259]. M^Pro^ is a dimer (306 residues) with two identical monomers, and is a significant target responsible for viral polyprotein cleavage 1ab at 11 (a major cleavage site), required for generating the NSP7–NSP8–NSP12 complex (viral replication complex) [260,261]. Residues interacting with two novel inhibitors against M^Pro^ have been identified: His41, Met49, Met165, Val186, Asp187, Arg188 as well as Gln189, exhibiting hydrophobic and H-bonding. Both inhibitors reside in the substrate-binding site and inhibit the enzymatic activity of M^Pro^ in SARS-CoV-2 [261].

Targeting highly conserved genes and/or proteins including RdRp (NSP12), M^Pro^, and helicases is a promising antiviral drug development approach to inhibit the replication and proliferation of SARS-CoV-2 [262]. Hence, inhibitors targeting these enzymes may reduce the threat of mutation-mediated drug resistance and facilitate effective antiviral protection [262]. A conserved motif (Ser-Aps-Asp) in the RdRp domain was identified at the C-terminus. The binding and activity of RdRp were enhanced by the NSP7–NSP8 (cofactor) complex. This binding stabilizes the entire closed conformation, which is packed beside the thumb–finger interface. The binding residues between the RdRp and RNA complex and RdRp docking to develop inhibitors have been extensively studied [78,79,80].

The inhibition of RdRP is important as one of the key strategies for developing antiviral therapeutics. The selective inhibition of RdRp may not cause noteworthy side effects or toxicity in host cells [263]. Natural compounds and their derivatives have exhibited significant binding affinity to RdRp [264,265,266], with promising outcomes that require further investigation.

NSPs 7–16 are responsible for coronavirus RNA synthesis and processing, which generate two large replicase polyproteins by cleavage. SARS-CoV-2 possesses a large number of enzymes that are responsible for RNA synthesis as well as RNA processing. The genome that is expressed and replicated by enzymes is two to three times larger than that of any other RNA viruses. RdRp is an important drug target because of its vital role in generating viral RNA [77,78,79,80].

Coronaviruses possess three important virulence factors: NSP1, NSP3c, and ORF7a. These factors help in the escape of viruses from host innate immunity and may be potential drug targets [55,267]. NSP1 and NSP3c interact with the host 40S ribosomal subunit and adenosine diphosphate-ribose, respectively. This leads to the degradation of mRNA and the inhibition of type-I IFN synthesis by NSP1, while NSP3c assists viruses to counterattack the immune response of the host [55,268]. ORF7a is directly attached to bone marrow matrix antigen-2 (BST-2) and has the ability to stop its glycosylation. BST-2 plays a specific role in regulating the release of newly synthesized viruses [267,269].

## 7. Conclusions

SARS-CoV-2 is recognized by various TLRs. Surface (TLR2 and 4) and intracellular (TRL3, 7/8, and 9) factors have been reported to be involved in the perception of SARS-CoV-2 infection by the immune system. Multiple adaptors such as MyD88 and TRIF are recruited by TLRs to initiate downstream signaling pathways. Various protein targets from viruses and the host machinery have been suggested as potential drug targets against SARS-CoV-2. Protein targets from viruses include both structural and nonstructural proteins. Similarly, TLRs are functional protein targets during SARS-CoV-2 infection.

## Figures and Tables

**Table 3:** Toll-like receptors (TLRs) and their physiological significance.

TLRs	Ligand Recognition	Form	Localization	Adaptor Molecules	Negative Adaptors	Response	Reference
TLR1	Triacyl lipopeptides, soluble factors	Heterodimer	Cell surface	MyD88, Mal	-	NF-κB activation and proinflammatory cytokines	[99,100]
TLR2	Hsp70, lipopeptide, HCV, Nonstructural protein 3	Heterodimer	Cell surface	MyD88, Mal	-	NF-κB activation and proinflammatory cytokines	[101,102]
TLR3	dsRNA	Homodimers	Endosomal membrane	TRIF	SARM negatively regulates TRIF	IRF activation, production of type 1 IFNs and proinflammatory cytokines	[103,104]
TLR4	Lipopolysaccharide, Taxol, S protein of SARS-CoV-2	Homodimers	Cell surface	MyD88, Mal, TRIF, TRAM	SARM negatively regulates TRIF and TRAM to consequently reduce inflammation	Activation of NF-κB, pro-inflammatory cytokines, and IFN-inducible genes	[105,106]
TLR5	Flagellin	Homodimers	Cell surface	MyD88	-	Activation of NF-κB and proinflammatory cytokines	[107,108]
TLR6	Diacyl lipopeptides, lipoteichoic acid, fungal zymosan	Heterodimer	Cell surface	MyD88, Mal/TIRAP	-	Activation of NF-κB and proinflammatory cytokines	[109,110]
TLR7	SARS-CoV-2 ssRNA, imadozoquinoline	Homodimers	Endosomal membrane	MyD88	-	IRF activation, production of Type 1 IFNs and proinflammatory cytokines	[111,112]
TLR8	SARS-CoV-2 ssRNA		Endosomal membrane	MyD88	-	IRF activation, production of type 1 IFNs and proinflammatory cytokines	[113,114]
TLR9	Unmethylated CPG-containing ssDNA, hemozoin from the malaria parasite	Homodimers	Endosomal membrane	MyD88	-	IRF activation, production of type 1 IFNs and proinflammatory cytokines	[115,116]

**Table 4:** Reported antiviral agonists employing Toll-like receptors (TLRs).

Drugs	TLRs	Viruses	Significance	References
Pam_2_CSK_4_	TLR2	Parainfluenza	Reduced virus replication	[141]
INNA-051	TLR2	SARS-CoV-2	Reduces viral RNA load	[142]
PIKA	TLR3	Influenza A	Reduces virus load	[143]
Poly ICLC	TLR3	HIV	Release of IFN-α/β/γ	[144]
NA6	TLR4	Norovirus	Induction of IFN-β	[145]
MPL	TLR4	VZV	Stimulate cytokines	[146]
Flagellin	TLR5	Influenza A	Reduces virus replication	[147]
CBLB502	TLR5	ConA	Activation of NF-κB	[148]
Pam_2_CSK_4_	TLR6	Parainfluenza	Reduces virus replication	[141]
INNA-051	TLR6	SARS-CoV-2	Reduces viral RNA load	[142]
GS-9620	TLR7	HIV	Reactivates latency	[112]
Vesatolimod	TLR7	HIV	Modest delay in viral rebound	[149]
R848	TLR7/8	Zika	Activation of NF-κB	[150]
GS-9688	TLR8	HBV	Activation of dendritic and natural killer cells	[151]
ODN2395	TLR9	Parainfluenza	Reduces viral replication	[141]

CBLB502—Entolimod; ConA—Concanavalin A; GS-9688—Selgantolimod; R848—Resiquimod; NA6—neoagarohexaose; VZV—Varicella-Zoster virus.

## Data Availability

Not applicable.
